# MTDeepM6A-2S: A two-stage multi-task deep learning method for predicting RNA N6-methyladenosine sites of *Saccharomyces cerevisiae*

**DOI:** 10.3389/fmicb.2022.999506

**Published:** 2022-10-05

**Authors:** Hong Wang, Shihao Zhao, Yinchu Cheng, Shoudong Bi, Xiaolei Zhu

**Affiliations:** School of Sciences, Anhui Agricultural University, Hefei, China

**Keywords:** N6-methyladenosine site, BiLSTM, post-transcriptional modification, computational methods, multi-task learning

## Abstract

N6-methyladenosine (m6A) is one of the most important RNA modifications, which is involved in many biological activities. Computational methods have been developed to detect m6A sites due to their high efficiency and low costs. As one of the most widely utilized model organisms, many methods have been developed for predicting m6A sites of *Saccharomyces cerevisiae*. However, the generalization of these methods was hampered by the limited size of the benchmark datasets. On the other hand, over 60,000 low resolution m6A sites and more than 10,000 base resolution m6A sites of *Saccharomyces cerevisiae* are recorded in RMBase and m6A-Atlas, respectively. The base resolution m6A sites are often obtained from low resolution results by post calibration. In view of these, we proposed a two-stage deep learning method, named MTDeepM6A-2S, to predict RNA m6A sites of *Saccharomyces cerevisiae* based on RNA sequence information. In the first stage, a multi-task model with convolutional neural network (CNN) and bidirectional long short-term memory (BiLSTM) deep framework was built to not only detect the low resolution m6A sites but also assign a reasonable probability for the predicted site. In the second stage, a transfer-learning strategy was used to build the model to predict the base resolution m6A sites from those low resolution m6A sites. The effectiveness of our model was validated on both training and independent test sets. The results show that our model outperforms other state-of-the-art models on the independent test set, which indicates that our model holds high potential to become a useful tool for epitranscriptomics analysis.

## Introduction

To date, more than 160 kinds of RNA post-transcriptional modifications (PTM) have been determined (Maden, 1990; Wang et al., 2014), among which N6-methyladenosine (m6A) is the most abundant internal mRNA modification (Liu and Jia, 2014). This modification was dynamically regulated by methyltransferase and demethylases (Desrosiers et al., 1974; Adams and Cory, 1975; Furuichi et al., 1975; Wei et al., 1975). More than 5,000 different mRNA molecules contain m6A, which implies that this modification could have a broad effect on gene expression patterns. It is reported that m6A plays important roles in various biological processes (Chen et al., 2015a), such as alternative splicing (Meyer et al., 2012), regulation of circadian rhythms (Fustin et al., 2013), cell differentiation and reprogramming (Aguilo et al., 2015; Vu et al., 2017), and primary microRNA processing (Ma et al., 2017), which are essential for cell development. Therefore, the accurate identification of m6A sites is a key step to understand the mechanisms of these biological phenomena.

Several experimental approaches have been developed to locate m6A sites. High-throughput sequencing technologies, such as MERIP (Meyer et al., 2012), M6A-Seq (Dominissini et al., 2012) and PA-M6A-Seq (Chen et al., 2015a), have been applied to detect m6A sites in various species, such as *Saccharomyces cerevisiae* (Schwartz et al., 2013), *Homo sapiens* (Dominissini et al., 2012; Linder et al., 2015), *Arabidopsis thaliana* (Luo et al., 2014) and *Mus musculus* (Dominissini et al., 2012). However, most high-throughput sequencing technologies cannot determine the exact location of the m6A sites (Chen et al., 2020). The m6A motif RRACH was often used to further narrow down the location of m6A sites to base-resolution within the peaks detected with m6A signal. Other experimental techniques such as miCLIP-seq (Linder et al., 2015) can identify the m6A sites at the single-nucleotide resolution level, however, these kinds of methods depend on m6A-specific antibodies and have poor reproducibility and complicated process. Recently, two RNA endoribonuclease based methods (Garcia-Campos et al., 2019; Zhang et al., 2019) have been developed to map m6A in single-base resolution, however, these methods still have their limitations as stated in Garcia-Campos et al.'s paper (Garcia-Campos et al., 2019). For example, the quantitative identification of m6A is only referred to a subset that occurred at ACA sites, the methylation deficient mutants are not always available, the distribution of insert lengths in the sequenced libraries can differ from one library to another and complicate between-sample analyses, and so on. Lately, Zhang et al. (2021) developed a method to systematically calibrate post-transcriptional modification sites by using a synthetic modification-free RNA library. The traditional time-consuming and expensive experimental methods are far from meeting the needs (Ditzler et al., 2015). Thus, fast computational methods would be helpful in identifying the m6A sites.

Until now, various computational methods have been developed to identify the m6A sites in different species. About 14, 7, 6, and 18 methods have been developed to predict m6A sites in *Homo sapiens* genome (Xiang et al., 2016a; Zhou et al., 2016; Chen et al., 2017, 2019; Xing et al., 2017; Qiang et al., 2018; Zhang and Hamada, 2018; Zhao et al., 2018; Nazari et al., 2019; Wu et al., 2019; Zou et al., 2019; Liu et al., 2020a,b; Li et al., 2021), *Mouse musculus* genome (Xiang et al., 2016a; Zhou et al., 2016; Chen et al., 2017; Qiang et al., 2018; Zhang and Hamada, 2018; Nazari et al., 2019; Zou et al., 2019), *Arabidopsis thaliana* genome (Chen et al., 2016; Xiang et al., 2016b; Xing et al., 2017; Huang et al., 2018; Qiang et al., 2018; Wang and Yan, 2018), and *Saccharomyces cerevisiae* genome (Chen et al., 2015b,c, 2017, 2018; Jia et al., 2016; Li et al., 2016; Liu et al., 2016; Zhang et al., 2016; Xing et al., 2017; Akbar and Hayat, 2018; Huang et al., 2018; Qiang et al., 2018; Wei et al., 2018, 2019; Nazari et al., 2019; Zhuang et al., 2019; Khan et al., 2020; Mahmoudi et al., 2020), respectively. These methods can be classified into two major categories, including the traditional machine learning- and deep learning-based methods. At the early stage, the machine learning algorithms were extensively leveraged to build different models. SRAMP (Zhou et al., 2016) is a model based on random forest to predict m6A sites of human and mouse. RFAthM6A (Wang and Yan, 2018) is another model based on random forest which is for *Arabidopsis thaliana*. iRNA-methyl (Chen et al., 2015b) is a model based on support vector machine (SVM) to detect m6A sites of *Saccharomyces cerevisiae*. In recent years, deep learning methods have been used to predict m6A sites. DeepM6ASeq (Zhang and Hamada, 2018) is a method based on convolutional neural network (CNN) and long short-term memory (LSTM) to identify m6A sites for human, mouse and zebra fish. iMethyl-Deep (Mahmoudi et al., 2020) is a model based on CNN to predict m6A sites of *Saccharomyces cerevisiae*.

As one of the most widely utilized model organisms, many methods (Chen et al., 2015b,c, 2017, 2018; Jia et al., 2016; Li et al., 2016; Liu et al., 2016; Zhang et al., 2016; Xing et al., 2017; Akbar and Hayat, 2018; Huang et al., 2018; Qiang et al., 2018; Nazari et al., 2019; Wei et al., 2019; Wu et al., 2019; Zhuang et al., 2019; Khan et al., 2020; Mahmoudi et al., 2020) have been developed for predicting m6A sites of *Saccharomyces cerevisiae*. However, these methods are mainly based on a small dataset which contains only 1,307 m6A sites that are based on base resolution sequencing, and the equal number of negative samples were selected randomly. Because of the limited size of the dataset, the power of deep learning methods cannot be fully exerted. However, over 60,000 low-resolution and 10,000 base resolution m6A sites of *Saccharomyces cerevisiae* have been recorded in RMBase (Sun et al., 2016; Xuan et al., 2018) and m6A-Atlas (Tang et al., 2021), respectively, which are not fully used for developing computational methods. As mentioned above, the high resolution m6A sites have been identified by post calibration. Inspired by these multi-stage processes for identifying high resolution m6A sites, we proposed a two-stage deep learning strategy to predict m6A sites of *Saccharomyces cerevisiae*.

In the first stage, we developed a multi-task deep learning method for detecting low-resolution m6A sites. To this end, the m6A sites and the corresponding “SupportNum” information recorded in RMBase were collected for classification and regression tasks, respectively. Multi-task learning algorithm has been used both for classification and regression (Cipolla et al., 2018; Vandenhende et al., 2022), which improves the learning effect of each task by sharing datasets, feature representation and other information and obtaining task-related output layers (Caruana, 1998). Because of its high efficiency and accuracy for dealing with related tasks, multi-task learning has been applied in biometric sites recognition. MTTFsite (Zhou et al., 2019) is a multi-task learning framework that includes a shared CNN to learn common features of all cell types. In MTDsite (Sun et al., 2021), a multi-task deep learning strategy was used to develop a novel sequence-based approach for simultaneous prediction of binding residues/sites for multiple important molecular types. However, multi-task learning has not been applied in the prediction of m6A sites.

In the second stage, the model was developed to identify the base resolution m6A sites from those low-resolution m6A sites predicted by the first stage model. So, we employed the base resolution m6A sites of *Saccharomyces cerevisiae* recoded in m6A-Atlas as positive samples and the low-resolution m6A sites recorded in RMBase as negative samples of which the duplicates to the sites of m6A-Atlas were removed. Considering the similarity between the classification tasks of the two stages, specific layers and weights of the deep network for the first stage model were transferred to build the second stage model.

## Materials and methods

### Datasets

To build a reliable deep learning predictor, the large-scale dataset is essential. For the first stage model, we collected low resolution m6A sites of *Saccharomyces cerevisiae* from an extensive database RMBase v2.0 (http://rna.sysu.edu.cn/rmbase/) (Xuan et al., 2018), in which 67,582 m6A sites were recorded. The RNA segments with *k* upstream and downstream nucleotides were obtained from the genome. Two types of central motif patterns exist in these segments which are AAC and GAC. Because the dataset Met2614 (Chen et al., 2015b) which was used to build the existing methods for predicting m6A sites of *Saccharomyces cerevisiae* contains only GAC central motif, we divided the original RNA segments into two parts: one contains the segments with GAC central motif and the other one contains the segments with AAC central motif. The numbers of segments with GAC and AAC central motifs are 23,581 and 44,001, respectively. The negative segments were collected from the genome which include 35,296 and 77,484 segments with central GAC and AAC motifs, respectively. Thus, we built two original datasets with central GAC and AAC motifs, respectively.

To determine the optimal segment length, we extracted segments with lengths of 51, 201, 401, 601, 801, 1,001, and 1,201 nt for the data with AAC central motif, respectively. The data with AAC central motif was used as the dataset to select the optimal segment length, because the data with AAC central motif is larger than the data with GAC central motif and the larger dataset would be better for training and optimizing a deep learning network. Supplementary Table S1 shows the parameters for each layer for optimizing the segment length. To avoid overfitting, CD-HIT (Fu et al., 2012) was used to remove redundant segments with a threshold of 0.7. The redundancies of positive and negative samples were removed, respectively. Then, the under-sampling was used to select the same number of negative samples as that of positive samples to ensure the balance of the datasets. Preliminary result shows that the segments with 601 nt achieved the best performance. Therefore, we selected the optimal sequence length of 601 nt for building our models.

By using the segments with 601 nt, the AAC dataset contains 13,732 negative samples and positive samples, respectively. And the GAC dataset contains 10,937 positive samples and negative samples, respectively. We named these two datasets as GAC21874 and AAC27464. The two datasets were randomly divided into training datasets and independent test datasets according to the ratio of 4:1, so that the training dataset of the GAC21874 contains 8,749 positive and negative samples, respectively. The independent test dataset contains 2,188 positive and negative samples, respectively. On the other hand, the training dataset of AAC27464 contains 10,985 positive and negative samples; the independent test dataset contains 2,747 positive and negative samples, respectively. Table 1 shows the datasets used in this study.

**Table 1:** Benchmark datasets demonstration.

	**Datasets**	**Positive samples**	**Negative samples**
Stage 1	GAC21874	10,937	10,937
	GAC_train	8,749	8,749
	GAC_test	2,188	2,188
	AAC27464	13,732	13,732
	AAC_train	10,985	10,985
	AAC_test	2,747	2,747
Stage 2	GAC_9378	4,689	4,689
	GAC_2344	1,172	1,172

**Figure 1:**
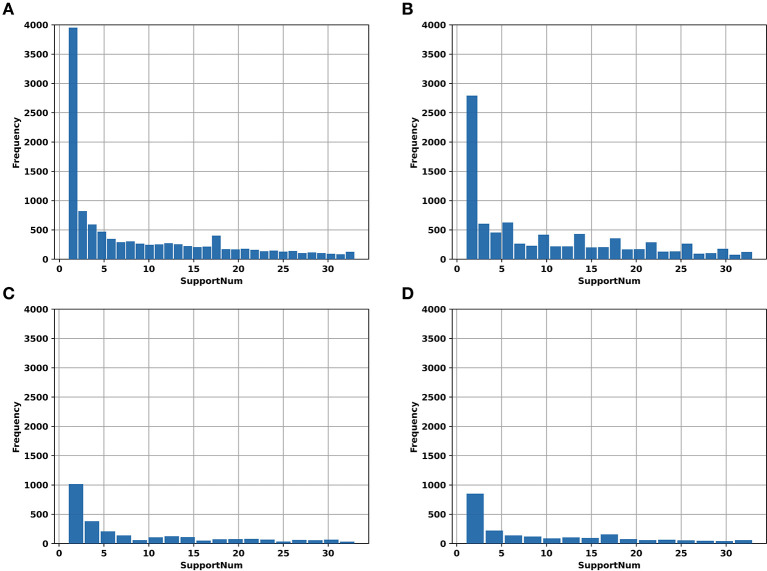
The histogram of “SupportNum” on different datasets. **(A)** AAC_train; **(B)** GAC_train; **(C)** AAC_test; **(D)** GAC_test.

In addition, the “SupportNum” for each m6A site recorded in RMBase v2.0 was used as the target for regression task. Although the m6A sites recorded in RMBase database can be used to train models for classification, the locations of m6A sites in the database were determined based both on the experimentally measured peak values and the RRACH motif. For each m6A site recorded in the database, the “SupportNum” represents how many experiments have demonstrated that the corresponding adenine can be modified. Intuitively, the more times the m6A site was identified by experiments, the higher confidence we have for the m6A site to be a realistic m6A site. Figure 1 shows the “SupportNum” distribution in our datasets. To take advantage of the information of “SupportNum” and give reasonable confidence for the predicted m6A sites of the classification model, we set a regression task in our multi-task learning architecture with the “SupportNum” as the target. Since the value range of “SupportNum” is relatively large, we first normalized it to [0,1]. Sklearn's Minmax Scaler was used for this normalization process.

For the second stage model, we collected base resolution m6A sites of *Saccharomyces cerevisiae* from m6A-Atlas (www.xjtlu.edu.cn/biologicalsciences/atlas) (Tang et al., 2021) as positive samples. Totally, 10,562 m6A sites were obtained, which are all with the central motif of GAC. Because the second stage model is used to identify base resolution m6A sites from low-resolution m6A sites, we used the low-resolution m6A sites recorded in RMBase 2.0 but not in m6A-Atlas as negative samples in the current study. Note, the negative samples were also with central motif of GAC. Thus, we obtained 10,562 positive samples and 14,810 negative samples. The redundancies of both positive and negative samples were removed by CD-HIT with a cutoff of 0.7. Thus, we got 5,861 positive samples and 8,924 negative samples. To obtain a balanced dataset, 5,861 negative samples were randomly selected to build the final dataset. The dataset was further divided into training and independent test set at a ratio of 4:1 (Table 1).

### Model construction

#### Overall framework of the two-stage model

**Figure 2:**
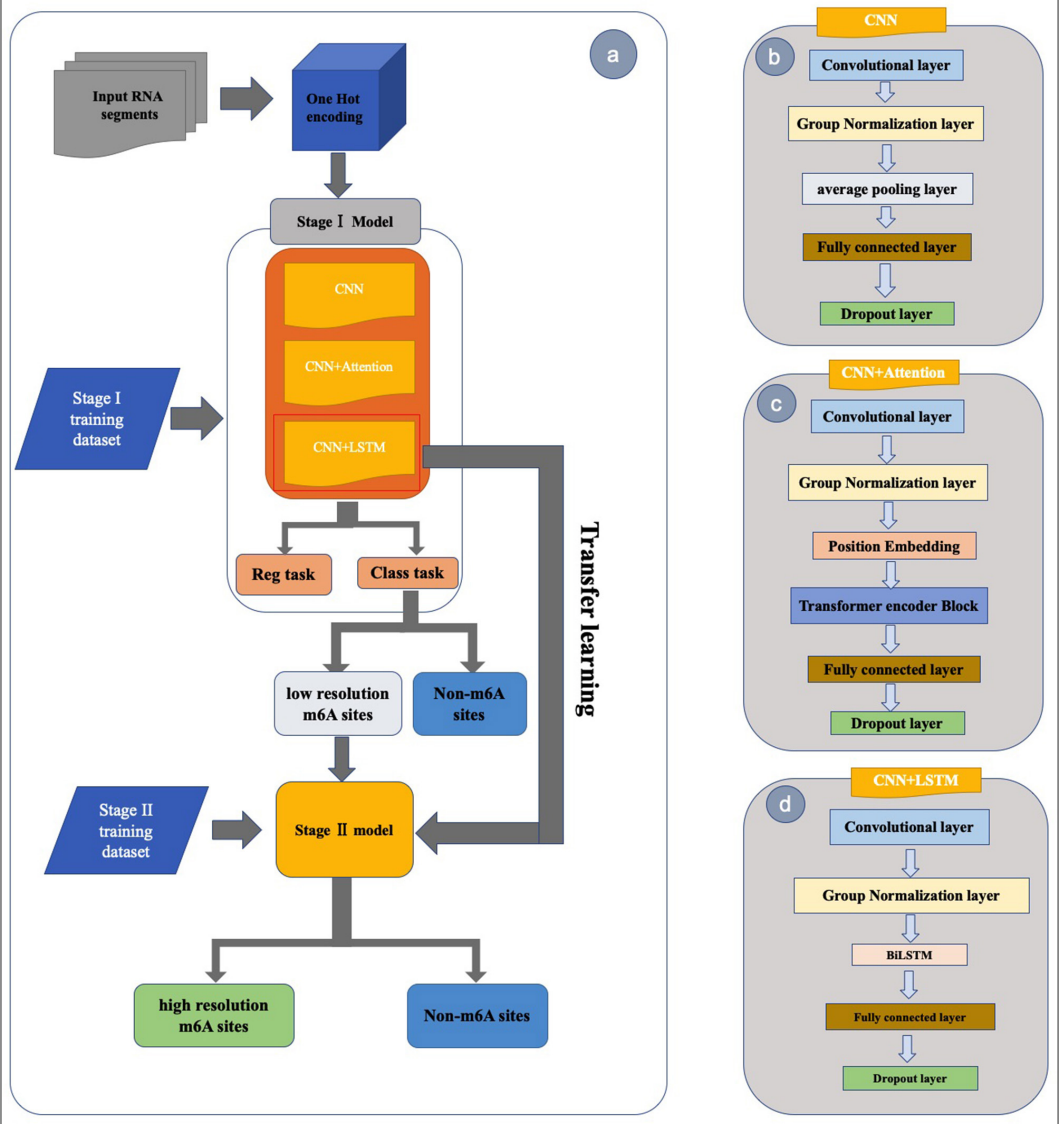
The overall flow chart of MTDeepM6A-2S. **(a)** The diagram of the two-stage model. The stage I model is used to discriminate low-resolution m6A sites from non-m6A sites and the stage II model is used to identify high-resolution m6A sites from low-resolution m6A sites. Three deep networks were used to build the stage I model which are **(b)** CNN network, **(c)** CNN+TRANSFORMER network and **(d)** CNN+LSTM network.

To fully use the existing experimental data and simulate the calibration process, we proposed a two-stage predictor. The flowchart for building our model is shown in Figure 2. The benchmark datasets collected from RMBase v2.0 were used to train and test our first stage model. A multi-task model was built in the first stage to classify the low resolution m6A sites and other non-m6A sites, which can also give a reasonable confidence for the classification result based on the regression task. For the second stage, we collected the data from m6A-Atlas to build our model which is used to identify the base resolution m6A sites from the low resolution m6A sites. The second-stage model was trained by using a transfer learning strategy based on the first-stage model.

#### The framework for the first stage model

The previous methods used to predict RNA m6A sites were mostly single-task learning for classification. However, we used a multi-task architecture to build the first-stage model. As shown in Figure 2a, the input for our model is the sequences of the RNA segments, then the sequences were transformed as numerical matrices by the one-hot encoding with A, U, C, G, N represented as (1,0,0,0), (0,1,0,0), (0,0,1,0), (0,0,0,1) and (0,0,0,0), respectively, where N is a padding pseudo nucleotide when the RNA segment is out of the genome. Then, the 4^*^601 matrix obtained by one-hot encoding was fed into the shared deep network of the multi-task model, and then the latent features encoded by shared deep networks were fed to the two task-specific networks. In this study, we compared three different shared deep networks which are CNN, CNN+BiLSTM and CNN+TRANSFORMER to select a better shared deep network. The three deep networks were explained as follows.

For the shared CNN network (Figure 2b) of the multi-task model, we used a 1D convolution layer to extract the global information of the sequence. The number of kernels was set to 16 but the size of the kernel used in the 1D CNN was optimized. Next, the output of the 1D CNN was normalized with a layer of Group normalization (GN) (Wu and He, 2018), for which the number of groups was set to 4.

For the shared CNN+BiLSTM network (Figure 2d) of the multi-task model, we added a BiLSTM layer based on the CNN network. The hidden unit of the cell of the BiLSTM was set as 8. BiLSTM overcomes the problem of not being able to retrieve information from upstream and downstream sequences.

For the shared CNN+TRANSFORMER network (Figure 2c) of the multi-task model, we added a position embedding layer and an Encoder of the Transformer (Vaswani et al., 2017) based on the CNN network. The Encoder is composed of a position embedding layer, a multi-head self-attention mechanism and a position-wise fully connected feed-forward network. The head number was set to 2. The “elu” activation function was used in all the three deep networks.

After the three deep networks, we used a 1D pooling layer to reduce the dimension of the features, the size of the kernel of the 1D pooling layer was optimized. After the pooling layer, a fully connected layer with 64 hidden nodes was added. To prevent overfitting, a dropout layer was added after the fully connected layer, and the dropout ratio was also optimized.

In the output layer, there are two outputs. For the classification task, the “softmax” activation function was used, and the categorical cross entropy was used as the loss function. For the regression task, the activation function “elu” was used and the “logcosh” was used as the loss function. Thus, the loss function of the whole multi-task can be expressed as follows:


$$\begin{array}{l} Loss_{multi}=-\frac{1}{N}\sum_{i=1}^{N}\left(y_{i}^{c}\log p_{i}^{c}+(1-y_{i}^{c}\right)\log\left(1-p_{i}^{c}\right)\\ +\;\sum_{i=1}^{N}\log(\cosh(y_{i}^{r}-{\hat{y}}_{i})), \end{array}$$

where, N represents the number of samples in the dataset, $y_{i}^{c}$ and $p_{i}^{c}$ mean the real label and predictive probability of the *ith* sample for the classification task, respectively. $y_{i}^{r}$ and ŷ_*i*_ show the real label and the predictive values of the *ith* sample for the regression task, and the former part of the multi-task loss (*Loss*_*multi*_) is the categorical cross entropy for the classification task, and the latter part is the log-cosh loss for the regression task. According to this loss function, the information of the label of the auxiliary task (regression task) might be able to help improve the predictive accuracy of the classification task.

At last, we used Stochastic Gradient Descent (SGD) as the optimization algorithm. Our deep learning network was built based on Keras version 2.2.4

#### Transfer learning for building the second stage model

Considering the similarity between the classification tasks of the two stages, we transferred the feature extraction layers of the first-stage model to build our second-stage model. Specifically, during transfer learning, the feature extraction layers (all layers except the output layer) and corresponding weights of the first-stage model were used as the initial parameters for the second-stage model and then all the weights of the second stage model were optimized without freezing in the training process.

To train our model, we needed to determine the network hyperparameters mentioned in Section Model construction such as dropout ratio, pool size and kernel size. We also needed to determine some general hyperparameters such as learning rate, batch size, and epochs. In this study, we used the module GridSearchCV of sklearn package to determine the hyperparameters of the first-stage model by 5-fold cross-validation. Supplementary Table S2 shows the ranges of hyperparameters that were optimized by GridSearchCV. Supplementary Table S3 shows the optimized values for the hyperparameters of the deep network.

### Evaluation criteria

To evaluate the performance of the predictors, we calculated four metrics which are sensitivity (Sn), specificity (Sp), accuracy (Acc), and Matthews correlation coefficient (MCC). The four parameters were defined as follows:


$$\{\begin{array}{c} \begin{array}{c} Sn=\frac{TP}{TP+FN}\;0<Sn<1\\ Sp=\;\frac{TN}{TN+\;FP}\;0<Sp<1\; \end{array}\\ \begin{array}{c} Acc=\;\frac{TP+TN}{TP+FP+TN+FN}\;0<Acc<1\\ \;MCC=\frac{\left(TP\times TN\right)-\left(FN\times FP\right)}{\sqrt{\left(TP+FN\right)\times \left(TN+FP\right)\times \left(TP+FP\right)\times \left(TN+FN\right)}}\\ \;-1<MCC<1 \end{array} \end{array}$$

where TP, TN, FP, and FN represent the number of true positive, true negative, false positive, and false negative, respectively. Sensitivity and specificity indicate the ratios of correctly predicted positive samples and negative samples, respectively. Accuracy represents the ratio of correctly predicted samples. The Matthews correlation coefficient is a balanced measurement index, which can be used even when the category data is unbalanced.

In addition, we plotted Receiver Operating Characteristic (ROC) curves and Precision-Recall curves (PRC). ROC curve is a comprehensive index to reflect the continuous variables of sensitivity and specificity, thus revealing the relationship between sensitivity and specificity. PRC reflects the balance between the accuracy of positive samples recognition and the coverage ability of the classifier. AUROC (area under ROC curve) and AUPRC (area under PRC curve) are the area under the ROC curve and the area under the precision-recall curve, respectively. The larger the AUROC and AUPRC are, the better the classifier is.

Pearson correlation coefficient is used as the index to evaluate the regression task, which can be calculated as follow:


$$r=\frac{\sum_{i=1}^{n}\left(X_{i}-\bar{X})(Y_{i}-\bar{Y}\right)}{\sqrt{\sum_{i=1}^{n}{\left(X_{i}-\bar{X}\right)}^{2}\sqrt{\sum_{i=1}^{n}{\left(Y_{i}-\bar{Y}\right)}^{2}}}}$$

where, *X*_*i*_ and *Y*_*i*_ represent the predicted target value and the actual target value of the sample *i*, respectively. Pearson correlation coefficient, also known as Pearson product-moment correlation coefficient, is a statistical quantity used to reflect the similarity degree of two variables. Its value range is [−1, 1], and when it equals 0, it indicates no correlation.

## Results

### Results for the first-stage model

**Figure 3:**
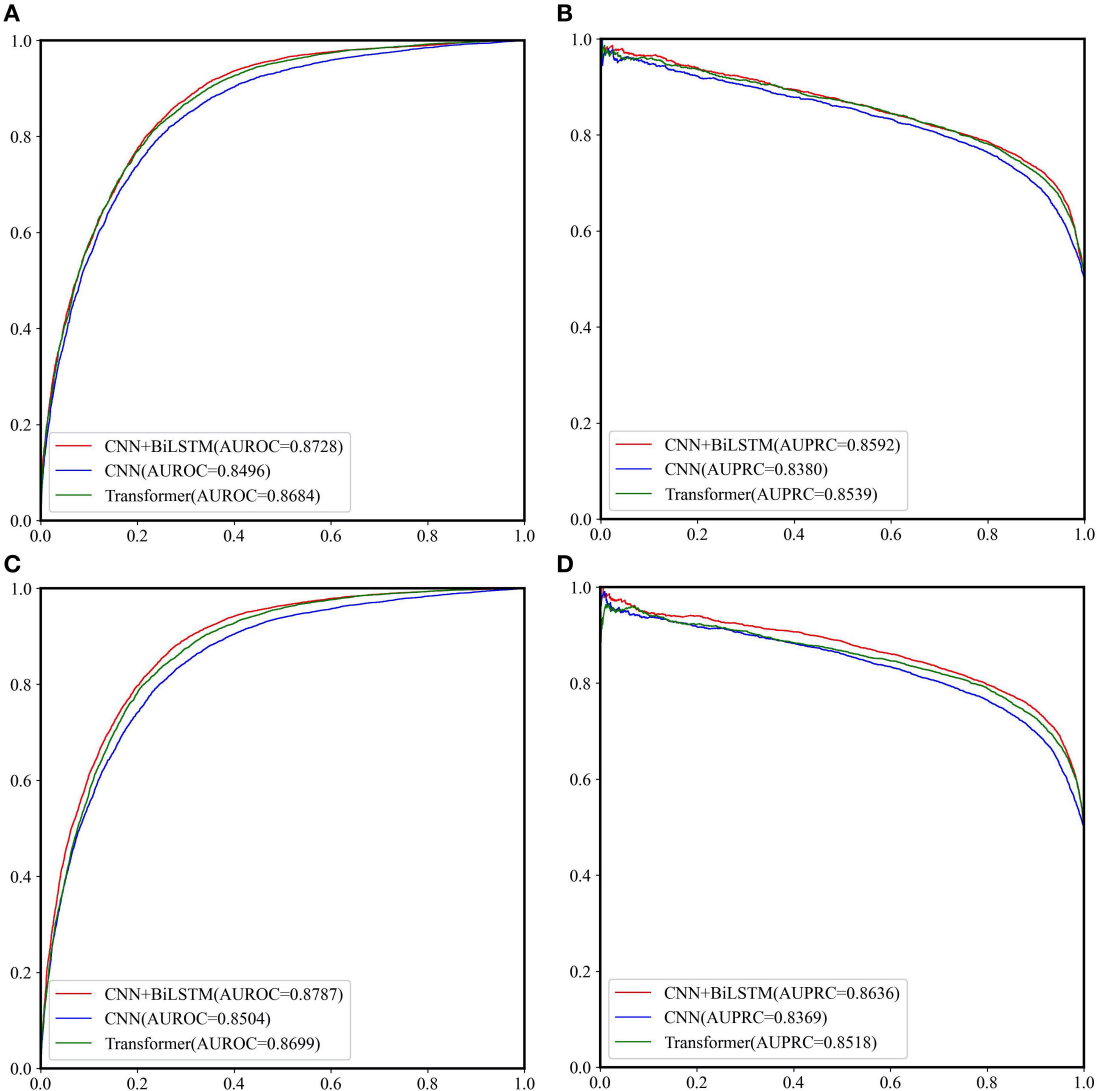
The ROC curves and PRC curves based on the cross-validation results of the three network frameworks on GAC_train and AAC_train. **(A)** ROC curves for GAC_train; **(B)** PRC curves for GAC_train; **(C)** ROC curves for AAC_train; **(D)** PRC curves for AAC_train.

The performance of CNN, CNN+BiLSTM and CNN+TRANSFORMER frameworks under multi-task learning was evaluated by 5-fold cross validation. As shown in Table 2 and Figure 3, for the classification task, the model with CNN+BiLSTM framework achieved the highest Acc, MCC, AUPRC, and AUROC for both GAC_train and AAC_train. The Acc, MCC, AUPRC and AUROC for GAC_train are 0.7919, 0.5869, 0.8592, and 0.8728, respectively, and the Acc, MCC, AUPRC and AUROC for AAC_train are 0.8002, 0.6019, 0.8636, and 0.8787, respectively. For the regression task, the model with CNN+BiLSTM framework also performed best, which achieved the highest Pearson correlation coefficient of 0.6186 and 0.6131 for GAC_train and AAC_train, respectively. The details of the predictive result for each fold based on CNN+BiLSTM framework are shown in Supplementary Tables S4–S6 for the three training datasets, respectively. Thus, the model with CNN+BiLSTM framework was selected as the final model for multi-task learning. We inferred that the CNN+BiLSTM model performs better than other models because we used a long sequence of RNA segment of 601 nucleotides. As shown in Tang et al.'s work (Tang et al., 2018), the bi-deep RNN-based model slightly outperforms CNN and Transformer models on modeling subject-verb agreement over long distances.

**Table 2:** Performance of three multi-task deep learning models on the two training datasets.

**Datasets**	**GAC_train dataset**			**AAC_train dataset**		
**Models**	**CNN+TRANSFORMER**	**CNN**	**CNN+BiLSTM**	**CNN+TRANSFORMER**	**CNN**	**CNN+BiLSTM**
Acc	0.7829	0.7735	0.7919	0.7930	0.7757	0.8002
Sp	0.6942	0.7792	0.7632	0.7573	0.7394	0.7705
Sn	0.8715	0.7679	0.8206	0.8286	0.8119	0.8299
MCC	0.5771	0.5480	0.5869	0.5894	0.5537	0.6019
AUPRC	0.8539	0.8380	0.8592	0.8518	0.8369	0.8636
AUROC	0.8684	0.8496	0.8728	0.8699	0.8504	0.8787
Corrcoef^a^	0.5994	0.5910	0.6186	0.5937	0.5901	0.6131

^a^Pearson correlation coefficient.

Table 3 shows the performance of CNN+BiLSTM model on GAC_test under multi-task learning. The model built based on GAC_train performs well on its test dataset with values of 0.7938, 0.6914, 0.8961, 0.6002, 0.8753, and 0.8740 for Acc, Sp, Sn, MCC, AUPRC, and AUROC, respectively. Moreover, the performance of the model established on AAC_train is superior to the model established on GAC_train on GAC_test. However, the difference is small.

**Table 3:** Performance of CNN+BiLSTM model on GAC_test under multi-task learning.

**Models**	**Acc**	**Sp**	**Sn**	**MCC**	**AUPRC**	**AUROC**	**Corrcoef**
AAC-CNN+BiLSTM*	0.7938	0.6914	0.8961	0.6002	0.8753	0.8842	0.6175
GAC-CNN+BiLSTM*	0.7947	0.6987	0.8906	0.6005	0.8652	0.8740	0.6077

AAC-CNN+BiLSTM^*^: The CNN+BiLSTM model built using AAC_train.

GAC-CNN+BiLSTM^*^: The CNN+BiLSTM model built using GAC_train.

Table 4 shows the performance of CNN+BiLSTM model on AAC_test under multi-task learning. The model established with AAC_train also performs well on its test dataset with values of 0.7968, 0.7143, 0.8793, 0.6019, 0.8698, and 0.8792 for Acc, Sp, Sn, MCC, AUPRC, and AUROC, respectively. Again, the model established on AAC_train has achieved better performance on AAC_test than the model established on GAC_train.

**Table 4:** Performance of CNN+BiLSTM model on AAC_test under multi-task learning.

**Models**	**Acc**	**Sp**	**Sn**	**MCC**	**AUPRC**	**AUROC**	**Corrcoef**
AAC- CNN+BiLSTM*	0.7968	0.7143	0.8793	0.6019	0.8698	0.8792	0.6056
GAC- CNN+BiLSTM*	0.7955	0.7266	0.8644	0.5967	0.8670	0.8752	0.5989

AAC-CNN+BiLSTM^*^: The CNN+BiLSTM model built using AAC_train.

GAC-CNN+BiLSTM^*^: The CNN+BiLSTM model built using GAC_train.

Tables 3, 4 indicate the model with CNN+BiLSTM framework trained on AAC_train outperforms the corresponding model trained on GAC_train for the two independent test sets. One of the possible reasons is that AAC_train is bigger than GAC_train. To validate this hypothesis, we built a model based on the combined dataset with both GAC_train and AAC_train. Table 5 shows that the performance of the model established on the combined dataset is superior to the model built on single training dataset according to the Acc, MCC, AUPRC, and AUROC values for classification and Pearson correlation coefficient values for regression. Supplementary Figure S1 shows the corresponding ROC curves and PRC curves.

**Table 5:** Performance of the CNN+BiLSTM model built on the combined dataset of GAC_train and AAC_train.

**Datasets**	**Acc**	**Sp**	**Sn**	**MCC**	**AUPRC**	**AUROC**	**Corrcoef**
Training dataset^a^	0.8076	0.7557	0.8594	0.6196	0.8738	0.8871	0.6369
GAC_test	0.7990	0.6892	0.9088	0.6130	0.8793	0.8867	0.6429
AAC_test	0.8052	0.7172	0.8931	0.6200	0.8789	0.8877	0.6338

^a^These are the cross validation results on the training dataset.

**Figure 4:**
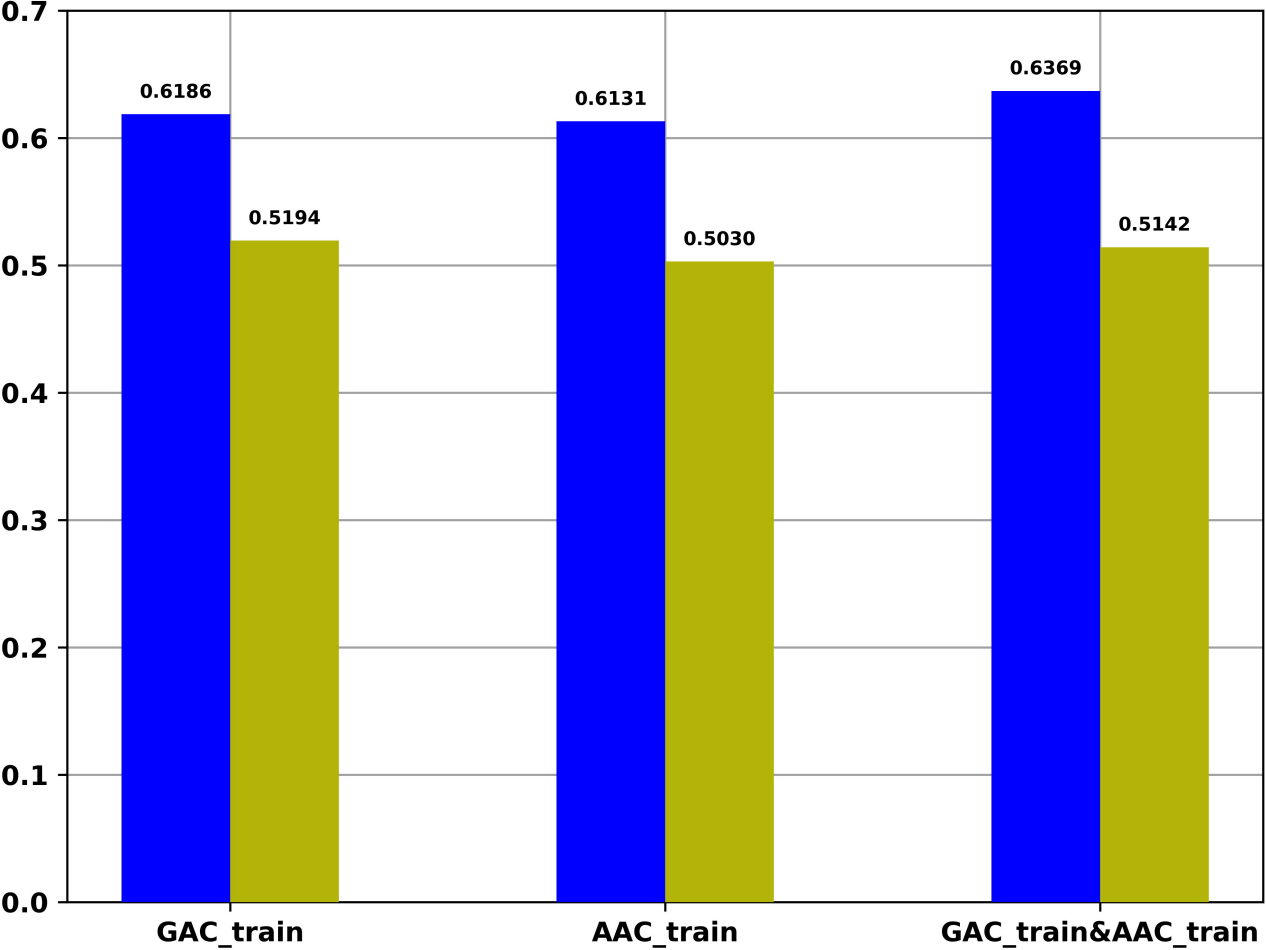
The Pearson correlation coefficient of multi-task models on different training datasets. Blue: The Pearson correlation coefficient obtained by regression task of multi-task models; Yellow: The Pearson correlation coefficient values obtained based on the predicted probability of the classification task of multi-task models and the target values of the regression task of multi-task models.

For the regression task, we compared the Pearson correlation coefficient obtained by our regression task with that obtained based on the predicted probability of the classification task and the target values of the regression task. Figure 4 shows the Pearson correlation coefficient values under different conditions. It indicates that the Pearson correlation coefficients obtained by our regression task are significantly better than the Pearson correlation coefficients obtained based on the predicted probability of the classification task.

### Results for the second-stage model

**Figure 5:**
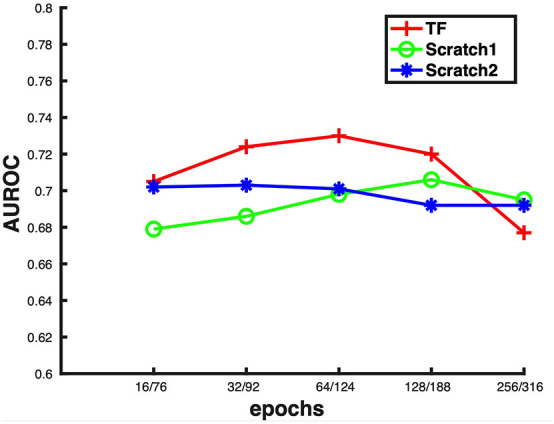
Cross-validation performances of three different learning strategies by using different epochs for the second-stage model. TF: Transfer learning; Scratch1: Learning from scratch with epochs from 16 to 256; Scratch2: Learning from scratch with epochs from 76 to 316.

Transfer learning was used to build the second-stage model based on the model built in the first stage. In this stage, the hyperparameter “epochs” were optimized to build our model whose range was from 16 to 256. Figure 5 shows the cross validation AUROCs of different epochs, which indicates the highest AUROC (0.7304) is obtained when epochs is 64. To investigate if the performance of transfer learning is superior to that of training from scratch, the same network without using the weights obtained from the first-stage model was trained. We set up two groups of epochs, one is from 16 to 256 and the other one is from 76 to 316 as the epochs used for building the first-stage model is 60. Figure 5 shows that the highest AUROCs obtained for the two groups of parameters are 0.7059 and 0.7030, respectively, which are lower than the AUROC obtained from transfer learning. Thus, our results demonstrate that transfer learning outperforms learning from scratch in this study. The details of the predictive result for each fold based on the final second-stage model are shown in Supplementary Table S7, and the ROC and PRC curves for the cross validation results are shown in Supplementary Figure S2.

### Comparison with existing predictors

**Figure 6:**
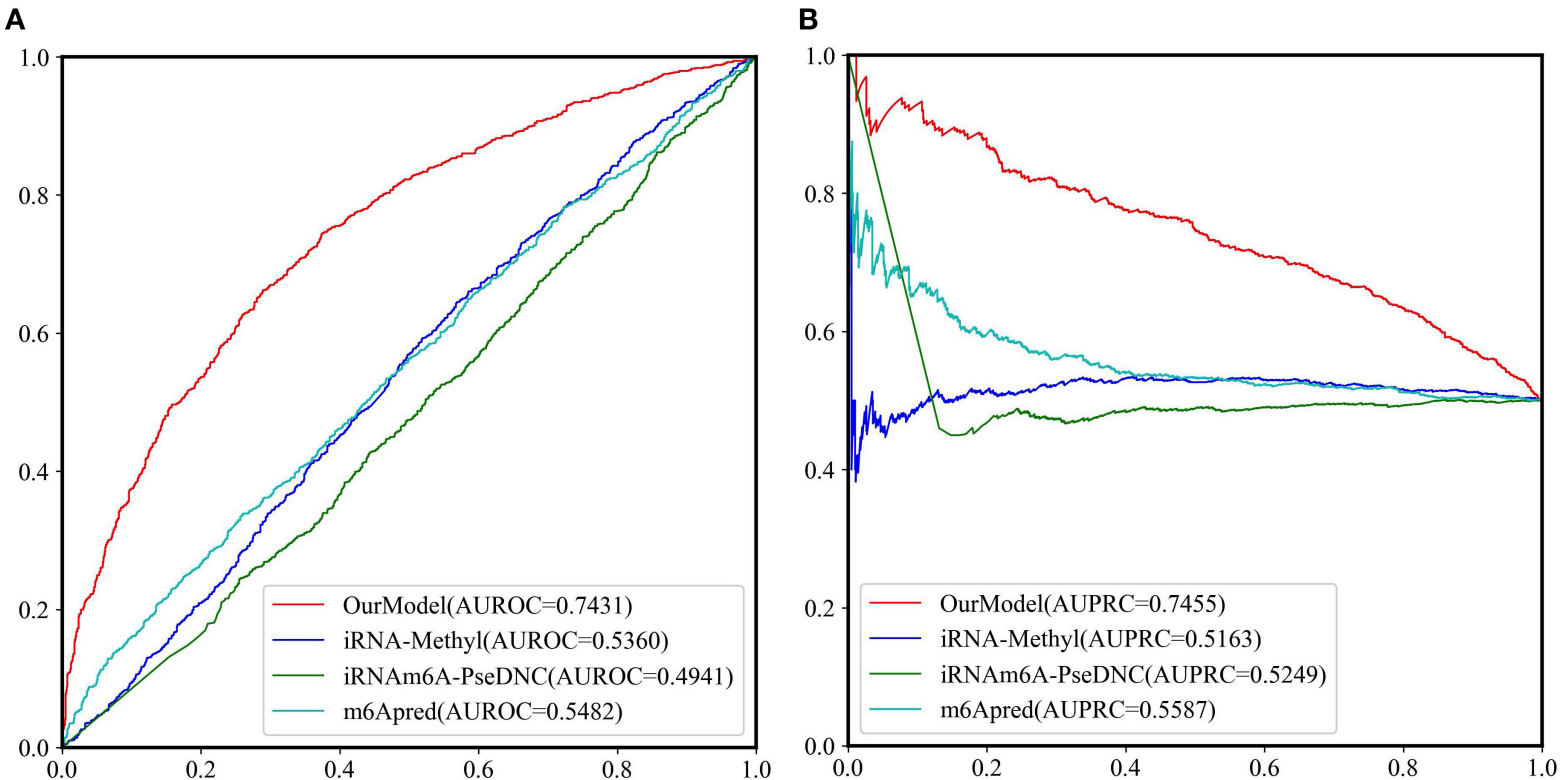
The ROC curves and PRC curves based on the predictive results of the independent test set GAC_2344 by our model and the other three methods. **(A)** ROC curves; **(B)** PRC curves.

Because most of the existing predictors were built based on the dataset which is composed of 1,307 base resolution data with GAC motif in the center, we decided to compare our second-stage model with these methods on the independent test set GAC_2344. Although several models such as BEMRP (Huang et al., 2018), m6Apred (Chen et al., 2015c), iRNA(m6A)-PseDNC (Chen et al., 2018) and iRNA-methyl (Chen et al., 2015b) have been developed to predict m6A sites of *Saccharomyces cerevisiae*. However, only m6Apred, iRNA(m6A)-PseDNC and iRNA-methyl are available now, Table 6 and Figure 6 show that our model substantially outperforms these three models on all the evaluation metrics except sensitivity. We noted that the model iRNA(m6A)-PseDNC even got a negative MCC. In the work for building the model iRNA(m6A)-PseDNC (Chen et al., 2018), the 1,307 negative samples were specifically selected according to the Euclidean distance (based on the 22 features) to the center of all 33,280 negative samples.

**Table 6:** Performance of different models on GAC_test.

**Models**	**Acc**	**Sp**	**Sn**	**MCC**	**AUPRC**	**AUROC**
iRNA-Methyl	0.5260	0.5350	0.5171	0.0521	0.5163	0.5360
M6Apred	0.5367	0.7509	0.3225	0.0812	0.5587	0.5482
iRNA(m6A)-PseDNC	0.4906	0.1681	0.8131	−0.0246	0.5249	0.4941
Our model	0.6813	0.7099	0.6527	0.3632	0.7455	0.7431

## Discussions

### Results of single-task models

In order to evaluate the efficacy of multi-task learning architecture, we conducted the two single-task learnings, i.e., classification task learning and regression task learning, based on the selected CNN+BiLSTM network, respectively. Table 7 shows the cross validation results of the single-task classification models on the two training datasets. According to AUROC, the performance of the multi-task model is a bit better than that of the single-task classification model on AAC_train, however, the performance of the multi-task model is a little worse than that of the single-task classification model on GAC_train. To further demonstrate the effectiveness of multi-task architecture, we also evaluated the performance on the dataset including both AAC_train and GAC_train. As shown in Table 7, the AUROC (0.8871), AUPRC (0.8738), MCC (0.6196) of the multi-task model are all better than that of the single-task classification model on this dataset.

**Table 7:** Performance comparison between the single-task classification models and the classification task of the multi-task models based on GAC_train, AAC_train, and GAC_train and AAC train, respectively.

**Datasets**	**GAC_train**		**AAC_train**		**GAC_train and AAC_train**	
**models**	**Single-task**	**Multi-task**	**Single-task**	**Multi-task**	**Single-task**	**Multi-task**
Acc	0.7928	0.7919	0.7997	0.8002	0.8069	0.8076
Sp	0.7608	0.7632	0.7840	0.7705	0.7872	0.7557
Sn	0.8248	0.8206	0.8154	0.8299	0.8267	0.8594
MCC	0.5877	0.5869	0.6000	0.6019	0.6153	0.6196
AUPRC	0.8598	0.8592	0.8605	0.8636	0.8711	0.8738
AUROC	0.8740	0.8728	0.8766	0.8787	0.8864	0.8871

Table 8 shows the cross validation results of the single-task regression models on AAC_train, GAC_train and the combined dataset of AAC_train and GAC_train. It shows that the performance of multi-task models is a little bit better than that of the single-task regression models on the three datasets according to the Pearson correlation coefficients.

**Table 8:** The cross-validation Pearson correlation coefficient of single-task regression models and multi-task models on GAC_train, AAC_train and GAC_train and AAC_train, respectively.

**Datasets**	**GAC_train**	**AAC_train**	**GAC_train and AAC_train**
Corrcoef of the regression task of multi-task models	0.6186	0.6131	0.6369
Corrcoef of the single-task regression models	0.6165	0.6070	0.6326

By comparing the performance between multi-task learning and single-task learning on the three datasets, GAC_train, AAC_train and the combined dataset. Our results show that the AUROCs of the classification-task are 0.8728 and 0.8740 based on multi-task learning and single-task learning for GAC_train, respectively, and the correlation coefficients of the regression task are 0.6186 and 0.6165 based on multi-task learning and single-task learning, respectively. On AAC_train, the AUROCs of the classification-task are 0.8787 and 0.8766 based on multi-task learning and single-task learning, respectively, and the correlation coefficients of the regression task are 0.6131 and 0.6070 based on multi-task learning and single-task learning, respectively. On the combined dataset with GAC_train and AAC_train, the AUROCs of the classification-task are 0.8871 and 0.8864 based on multi-task learning and single-task learning, respectively, and the correlation coefficients of the regression task are 0.6369 and 0.6326 based on multi-task learning and single-task learning, respectively. Thus, the multi-task learning outperforms the single-task learning for 5 of the 6 tasks on the three datasets. In addition, the multi-task learning framework is more efficient than single-task model because it can complete the two tasks simultaneously. Overall, our results indicate that the multi-task model slightly outperforms the single-task models.

### Performance of the first-stage model based on different loss weights and task-specific networks

To evaluate the effectiveness of different loss weights on our first-stage model, we used the strategy developed in Cipolla et al.'s work (Cipolla et al., 2018) to optimize the weights by using uncertainty and obtained a weights ratio of 0.06:1.85. Based on these weights, we validated the model based on AAC dataset and obtained a 5-fold cross validation AUROC of 0.8517, which is less than the value (0.8787) obtained based on the weight ratio of 1.0:1.0.

**Figure 7:**
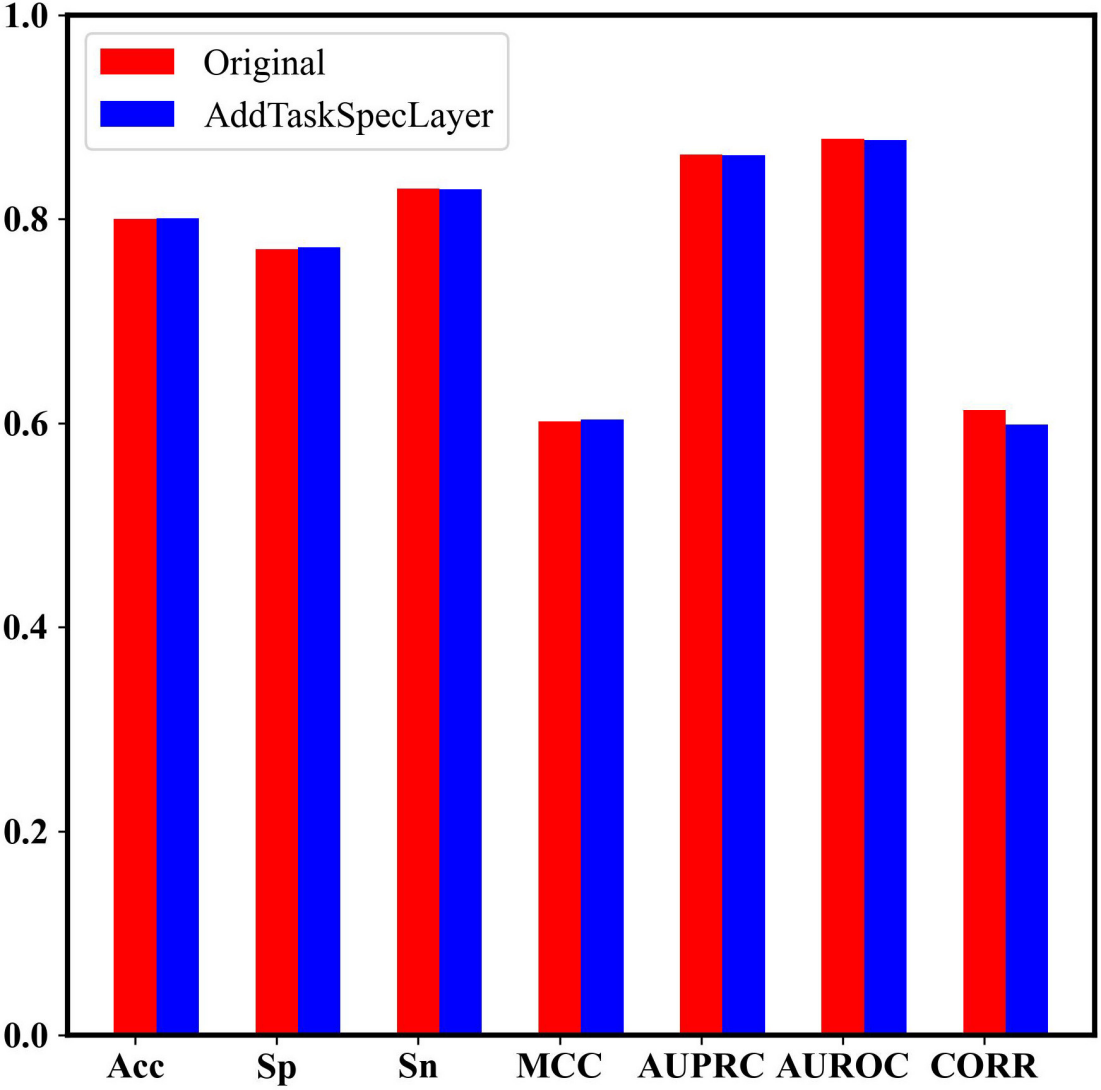
The performance comparison between the model with task-specific dense network and our original model.

In addition, we set up two dense layers with 64 nodes as the task-specific networks for the two tasks, respectively, to check if the performance could be improved. Figure 7 shows that the model with task-specific dense network achieves similar results to our original model.

### Analysis of sequences in the benchmark datasets of the two stages

Table 2 and Figure 5 show the AUROC of the first-stage model is higher than the second stage model, one possible reason is the discrepancy between positive and negative samples in the benchmark datasets of the two stages is different. Supplementary Figure S3 shows the graphical sequence logo generated by Two Sample Logo (Vacic et al., 2006) for the two benchmark datasets, which indicates the discrepancy between positive and negative samples of the training dataset of the first stage (upper panel) is larger than that of the second stage (lower panel). Thus, a two-stage model would be helpful for predicting m6A sites.

## Conclusions

In this study, we built a two-stage model, MTDeepM6A-2S, which can serially predict low resolution and base resolution m6A sites of *Saccharomyces cerevisiae* in the first and second stages. We used both the extensive low resolution data and the less extensive base resolution data to build our model, so as to simulate the process of wet experiments in which the base resolution m6A sites can be identified by post-calibration. To use the “SupportNum” information recorded in RMBase, we adopted a multi-task learning algorithm to build our first-stage model through which we can not only predict m6A sites of *Saccharomyces cerevisiae* but also give a reasonable confidence for the predictive results. Three deep learning networks were tried in this study, which are based on CNN, CNN+BiLSTM and CNN+TRANSFORMER frameworks. Our results indicate that the model with CNN+BiLSTM framework achieves the best performance. Further analysis also shows that the model based on multi-task learning is superior to the single-task learning. In the light of the similarity between the classification tasks of the two stages, transfer learning was employed to build the second-stage model so that the network weights obtained from the first stage could be used. In addition, the generalization of our model was evaluated on an independent test dataset, which indicates our model is substantially superior to the existing predictor, so that our model could be a useful tool for the community.

## Data availability statement

Publicly available datasets were analyzed in this study. This data can be found here: https://github.com/zhu313/MTDeepM6A-2S.

## Author contributions

XZ and SB conceived the study. XZ and HW designed the experiments. HW, SZ, and YC performed the experiments, analyzed the data, and wrote the article. All authors have read and agreed to the published version of the manuscript.

## Funding

This work was supported in part by National Natural Science Foundation of China (Grant Number: 21403002).

## Conflict of interest

The authors declare that the research was conducted in the absence of any commercial or financial relationships that could be construed as a potential conflict of interest.

## Publisher's note

All claims expressed in this article are solely those of the authors and do not necessarily represent those of their affiliated organizations, or those of the publisher, the editors and the reviewers. Any product that may be evaluated in this article, or claim that may be made by its manufacturer, is not guaranteed or endorsed by the publisher.
