# Supplementary material for: MTDeepM6A-2S: A two-stage multi-task deep learning method for predicting RNA N6-methyladenosine sites of Saccharomyces cerevisiae
Source: Front Microbiol. 2022 Oct 5;13:999506. doi: 10.3389/fmicb.2022.999506 (PMC9579691; doi:10.3389/fmicb.2022.999506)
Supplement: Supplementary file 1 [file Data_Sheet_1.docx]

Supplementary Materials for

**MTDeepM6A-2S: a two-stage multi-task deep learning method for predicting RNA N6-methyladenosine sites of *Saccharomyces cerevisiae***

**Table S1. The parameters for each layer of the CNN+TRANSFORMER network for optimizing the segment length**

| layers | Parameters | Values |
| --- | --- | --- |
| 1D convolution layer | filters | 16 |
|  | kernel size | 10 |
|  | activation function | ELU |
| GN layer | group number | 4 |
| Encoder of transformer | the number of headers in multi-head attention | 2 |
|  | output dimension of each header | 8 |
|  | pool size of the pooling layer after transformer | 15 |
| flatten layer | node number | 64 |
|  | activation function | ELU |
| dropout layer | dropout ratio | 0.6 |
| output layer | node number | 2 |
|  | activation function | Softmax |

**Table S2. Ranges of hyperparameters that were optimized by GridSearchCV.**

| Parameters | kernel_size | pool_size | dropout rate | learning rate | batch_size | epochs |
| --- | --- | --- | --- | --- | --- | --- |
| Ranges | [5,10,15] | [5,10,15] | [0.4,0.5,0.6] | [0.001,0.01,0.1] | [128,256] | [60,80,100] |

**Table S3. The optimized values for the hyperparameters of the deep network.**

| Parameters | kernel_size | pool_size | dropout rate | learning rate | batch_size | epochs |
| --- | --- | --- | --- | --- | --- | --- |
| Optimized values | 10 | 15 | 0.6 | 0.01 | 256 | 60 |

**Table S4.** **The cross-validation result for each fold of dataset GAC_train based on the CNN+BiLSTM model of the first stage.**

| Folds | Fold1 | Fold2 | Fold3 | Fold4 | Fold5 | average |
| --- | --- | --- | --- | --- | --- | --- |
| Acc | 0.7957 | 0.7900 | 0.7953 | 0.7885 | 0.7899 | 0.7919 |
| Sp | 0.7242 | 0.7020 | 0.7987 | 0.7885 | 0.8027 | 0.7632 |
| Sn | 0.8672 | 0.8781 | 0.7919 | 0.7885 | 0.7771 | 0.8206 |
| MCC | 0.5976 | 0.5893 | 0.5907 | 0.5770 | 0.5800 | 0.5869 |
| AUPRC | 0.8590 | 0.8661 | 0.8543 | 0.8575 | 0.8592 | 0.8592 |
| AUROC | 0.8721 | 0.8734 | 0.8719 | 0.8737 | 0.8727 | 0.8728 |
| Corrcoef^a^ | 0.6263 | 0.6109 | 0.6154 | 0.6189 | 0.6215 | 0.6186 |

^a^ Pearson correlation coefficient

**Table S5.** **The cross-validation result for each fold of dataset AAC_train based on the CNN+BiLSTM model of the first stage.**

| Folds | Fold1 | Fold2 | Fold3 | Fold4 | Fold5 | average |
| --- | --- | --- | --- | --- | --- | --- |
| Acc | 0.7844 | 0.7985 | 0.8051 | 0.8057 | 0.8073 | 0.8002 |
| Sp | 0.7383 | 0.8010 | 0.7528 | 0.7700 | 0.7905 | 0.7705 |
| Sn | 0.8305 | 0.7960 | 0.8573 | 0.8414 | 0.8241 | 0.8299 |
| MCC | 0.5713 | 0.5970 | 0.6135 | 0.6129 | 0.6149 | 0.6019 |
| AUPRC | 0.8540 | 0.8660 | 0.8729 | 0.8635 | 0.8619 | 0.8636 |
| AUROC | 0.8672 | 0.8812 | 0.8842 | 0.8801 | 0.8808 | 0.8787 |
| Corrcoef^a^ | 0.6011 | 0.6231 | 0.6173 | 0.6051 | 0.6189 | 0.6131 |

^a^ Pearson correlation coefficient

**Table S6.** **The cross-validation result for each fold of GAC_train & AAC_train based on the CNN+BiLSTM model of the first stage.**

| Folds | Fold1 | Fold2 | Fold3 | Fold4 | Fold5 | average |
| --- | --- | --- | --- | --- | --- | --- |
| Acc | 0.8073 | 0.8068 | 0.8120 | 0.8046 | 0.8071 | 0.8076 |
| Sp | 0.7439 | 0.7985 | 0.7861 | 0.7191 | 0.7310 | 0.7557 |
| Sn | 0.8708 | 0.8152 | 0.8379 | 0.8900 | 0.8832 | 0.8594 |
| MCC | 0.6197 | 0.6137 | 0.6249 | 0.6182 | 0.6214 | 0.6196 |
| AUPRC | 0.8677 | 0.8777 | 0.8717 | 0.8765 | 0.8752 | 0.8738 |
| AUROC | 0.8819 | 0.8887 | 0.8876 | 0.8890 | 0.8882 | 0.8871 |
| Corrcoef^a^ | 0.6252 | 0.6310 | 0.6431 | 0.6454 | 0.6397 | 0.6369 |

^a^ Pearson correlation coefficient

**Table S7.** **The cross-validation result for each fold of GAC_9378 based on the second-stage model.**

| Folds | Fold1 | Fold2 | Fold3 | Fold4 | Fold5 | average |
| --- | --- | --- | --- | --- | --- | --- |
| Acc | 0.6743 | 0.6754 | 0.6578 | 0.6727 | 0.6708 | 0.6702 |
| Sp | 0.6994 | 0.6695 | 0.6173 | 0.7324 | 0.7204 | 0.6878 |
| Sn | 0.6493 | 0.6812 | 0.6983 | 0.6130 | 0.6211 | 0.6526 |
| MCC | 0.3491 | 0.3508 | 0.3166 | 0.3479 | 0.3432 | 0.3415 |
| AUPRC | 0.7183 | 0.7405 | 0.7065 | 0.7149 | 0.7350 | 0.7230 |
| AUROC | 0.7294 | 0.7387 | 0.7149 | 0.7305 | 0.7384 | 0.7304 |


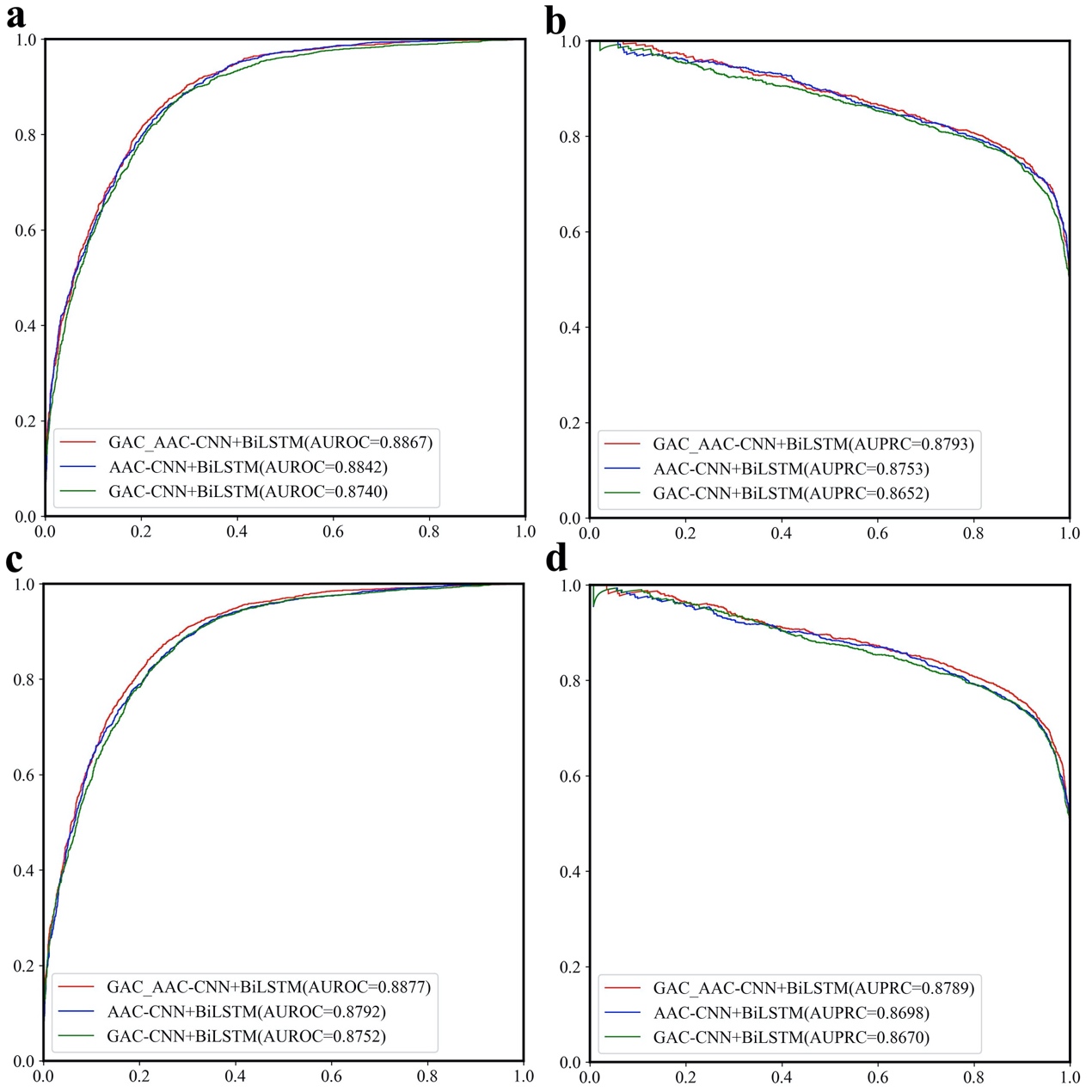


**Figure S1.** The ROC curves and PRC curves based on the predictive results on the independent test set GAC_test and AAC_test by the models built on three different datasets (GAC_train, AAC_train and GAC_train & AAC_train) with CNN+BiLSTM framework. a: ROC curves for GAC_test; b: PRC curves for GAC_test; c: ROC curves for AAC_test; b: PRC curves for AAC_test.

**
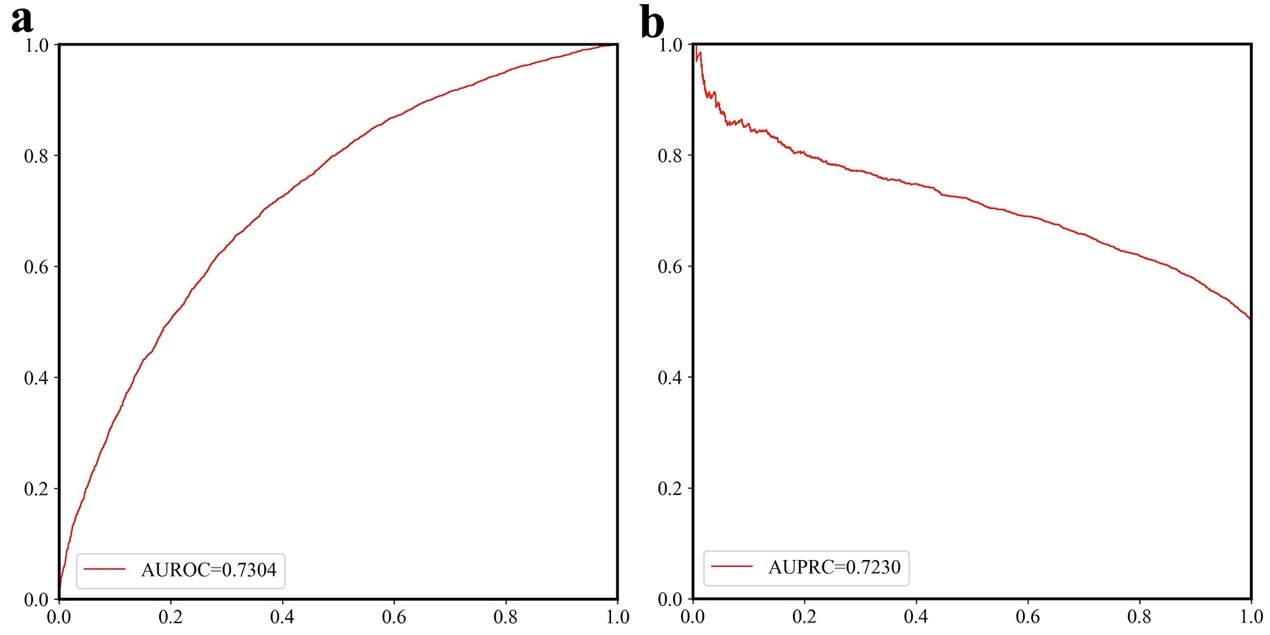
**

**Figure S2.** The ROC curve and PRC curve based on the cross-validation results of the second-stage model. a: ROC curve; b: PRC curve.

**
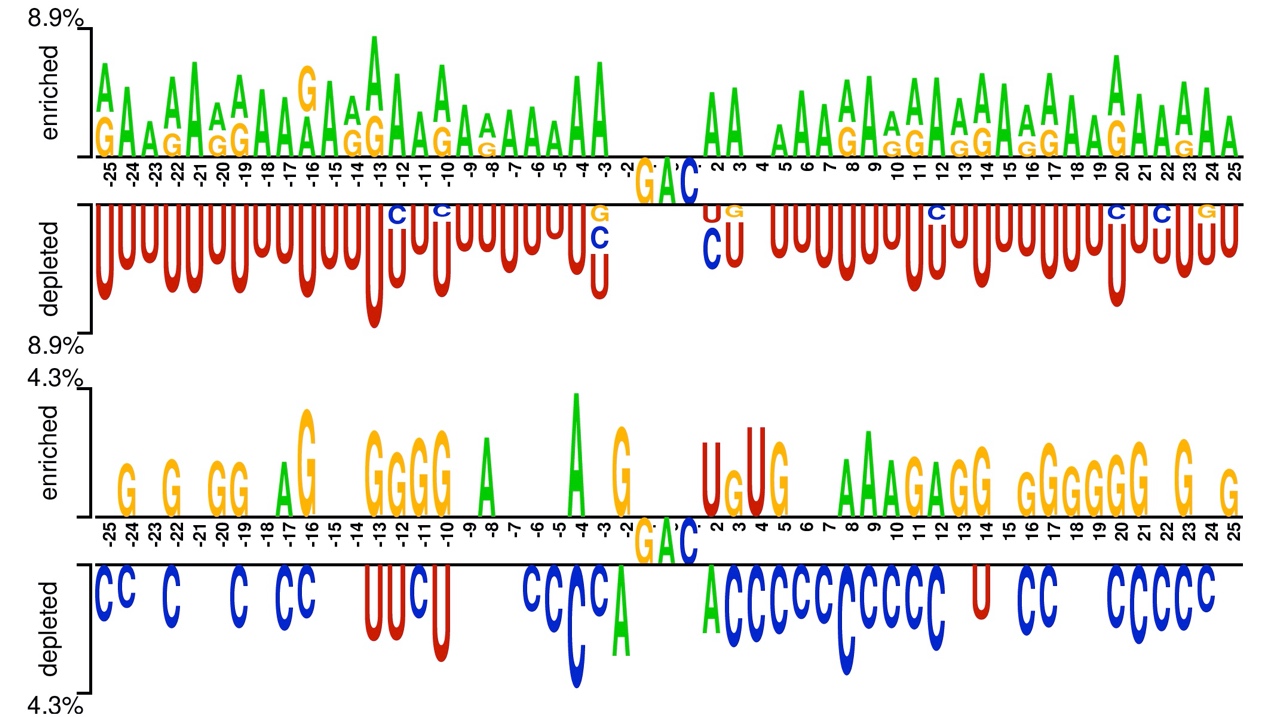
**

**Figure S3. Differences of sequence patterns between positive samples with negative samples. Upper panel: GAC_train; Lower panel: GAC_9378.**
